# Functional Diversity of Anti-Lipopolysaccharide Factor Isoforms in Shrimp and Their Characters Related to Antiviral Activity

**DOI:** 10.3390/md13052602

**Published:** 2015-04-27

**Authors:** Shihao Li, Shuyue Guo, Fuhua Li, Jianhai Xiang

**Affiliations:** 1 Key Laboratory of Experimental Marine Biology, Institute of Oceanology, Chinese Academy of Sciences, 7 Nanhai Road, Qingdao 266071, China; E-Mails: lishihao@qdio.ac.cn (S.L.); guoshuyue@126.com (S.G.); jhxiang@qdio.ac.cn (J.X.); 2 National & Local Joint Engineering Laboratory of Ecological Mariculture, 7 Nanhai Road, Qingdao 266071, China; 3 Graduate University of Chinese Academy of Sciences, Beijing 100049, China

**Keywords:** anti-lipopolysaccharide factor, LPS-binding domain, antibacterial, antiviral, lysine residue

## Abstract

Anti-lipopolysaccharide factor (ALF) is a small protein with broad-spectrum antimicrobial activity, which has potential application in the disease control. Previously, we isolated seven ALF isoforms from the Chinese shrimp *Fenneropenaeus* *chinensis*. In the present study, their distributions in tissues of shrimp were analyzed and the data showed that different isoforms had different expression profiles, which suggested that they might have different functions. Then, the functions of different isoforms were studied by analyzing the antibacterial and antiviral activities of the functional domain of ALFs, the LPS-binding domain (LBD), which were synthesized by chemical methods. Different ALFs showed distinct antibacterial and antiviral activities, which were consistent with their diverse tissue distribution patterns. Sequence analysis on the LBD domain of different isoforms revealed that an identical lysine residue site was specifically conserved in peptides with anti-WSSV activity. In order to confirm whether this lysine residue is critical to the antiviral activity of the peptide, new peptides were synthesized by changing residues at this site. Changing the lysine residue at the specific site to other amino acid residue, the antiviral activity of the peptide apparently decreased. While replacing other residue with a lysine residue at this site in LBD peptide without anti-WSSV activity, the peptide will obtain the antiviral activity to WSSV. These results not only showed us a comprehensive understanding on the function of ALFs from *F. chinensis*, but also provided clues for the development of ALFs as potential therapeutic drugs to WSSV.

## 1. Introduction

Anti-lipopolysaccharide factor (ALF) is a small protein with broad-spectrum antimicrobial activity, which has potential application in the disease control of shrimp. In the early 1980s, ALF was first isolated from hemocytes of horseshoe crab with the ability to bind and neutralize lipopolysaccharide (LPS) [1,2]. Until now, ALFs have been identified and characterized in different species of crustaceans.

ALFs have been verified responsive to different pathogen challenges and with multiple antimicrobial activities. *LvALF* from *Litopenaeus* *vannamei*, *PmALF* from *Penaeus* *monodon*, *EsALF* from *Eriocheir* *sinensis*, and *PtALF* from *Portunus* *trituberculatus*, have been reported to be expressed constitutively in hemocytes and up-regulated after bacteria challenges, which suggest that ALFs might play important roles in defense against bacterial pathogens [3,4,5,6,7,8]. The recombinant proteins of ALF genes by prokaryotic expression system exerted strong antibacterial effects on both Gram-positive and Gram-negative bacteria [4,5,8,9]. The recombinant ALF from *P. monodon* also showed antiviral activities against herpes simplex virus type 1 and human adenovirus respiratory strain in cultured mammalian cell lines [10] and white spot syndrome virus (WSSV) in cultured hematopoietic tissue (Hpt) cells from crayfish [11]. The expression of envelope protein VP28 of WSSV could be enhanced in the cultured Hpt cells from crayfish *Pacifastacus* *leniusculus* after ALF gene was interfered, and the mortality of LvALF1 knockdown shrimp increased significantly after *V. penaeicida*, *Fussarium* *oxysporum* or WSSV infections [3,12].

Different ALFs have a conserved cluster of positively charged residues within their disulfide loop between two conserved cysteine residues, which is usually called lipopolysaccharide (LPS)-binding domain [13,14,15]. The typical structure has been reported to be closely associated with the biological activities of ALFs and considered to be the vital functional domain [3,7,13,16,17,18,19]. The synthetic peptides corresponding to these LPS-binding domains, such as ALFSp, SsALF from *Scylla paramamosain* and *Scylla serrata* [17,20], and SALF_55–76_ from *P. monodon* [21] showed antibacterial activity against various bacteria. The synthetic LPS-binding domain from crayfish ALF could inhibit the replication of WSSV in cultured Hpt cells [11].

In our previous studies, the synthetic LBD peptides of two FcALF isoforms (*FcALF2* and *ALFFc*) were also found to inhibit bacterial growth and WSSV *in vivo* replication [22,23]. Further structure-activity analysis showed that their antibacterial activities were closely related with the disulfide bonds and basic amino acid residues in LBD peptides, while their anti-WSSV activities were associated with lysine residues [22,23]. In crustaceans, several ALF isoforms always coexist in one organism. In *P. monodon*, six ALF isoforms were identified by EST approach [7]. In *P. trituberculatus*, seven ALF isoforms were cloned and they displayed different biological activities [4,5,6,24,25]. Seven ALF isoforms (FcALF1-6 and ALFFc) were also identified in the Chinese shrimp *Fenneropenaeus chinensis* [26,27]. However, a comprehensive understanding on the diverse functions of these FcALFs is still very limited. In the present study, the functions of different FcALFs will be analyzed comparatively. These data will not only provide us a comprehensive understanding on the function of ALFs, but also give an instruction for the development of therapeutic drugs to shrimp disease control.

## 2. Results

### 2.1. Tissue Distribution of Different FcALFs Transcripts

**Figure 1:**
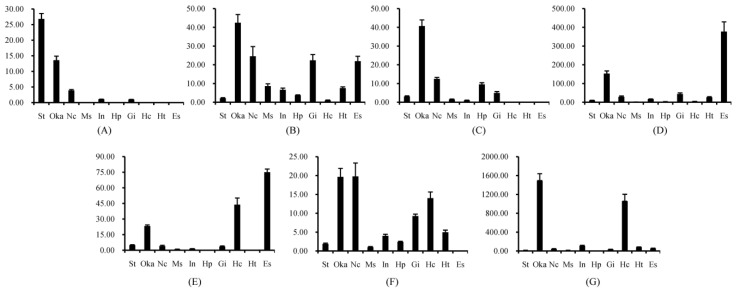
The tissue distribution of different *FcALFs* transcripts. *FcALF1*, *FcLAF2*, *FcLAF3*, *FcLAF4*, *FcLAF5*, *FcLAF6* and *ALFFc* were shown in (**A**–**G**), respectively. St, Stomach; oka, lymphoid organ; Nc, nerve cord; Ms, muscle; In, intestine; Hp, hepatopancreas; Gi, gill; Hc, haemocytes; He, heart; Es, eyestalk. Vertical bars represented the mean ± standard error (SE) (*n* = 10).

In order to have a basic understanding on their potential function, tissue distribution of *FcALFs* was performed. Different *FcALFs* showed their various expression profiles. *FcALF1* was prominently expressed in stomach, followed by Oka. Remarkably lower expression levels were detected in nerve cord, intestines, and gill. No expression was detected in muscle, hepatopancreas, hemocytes, heart, and eyestalk (Figure 1A). *FcALF2* was mainly expressed in Oka, followed by nerve cord, gill, and eyestalk (Figure 1B). *FcALF3* was also mainly expressed in Oka (Figure 1C). Both *FcALF4* and *FcALF5* were mainly detected in eyestalk and also showed high expression levels in Oka (Figure 1D,E), while *FcALF5* was also highly expressed in hemocytes (Figure 1E). *FcALF6* was detected in all tested tissues. The relatively high expression levels of *FcALF6* were present in Oka, nerve cord, hemocytes and gill (Figure 1F). *ALFFc* was mainly detected in Oka and hemocytes (Figure 1G).

### 2.2. Antibacterial Activities of Synthetic LBD Peptides

**Figure 2:**
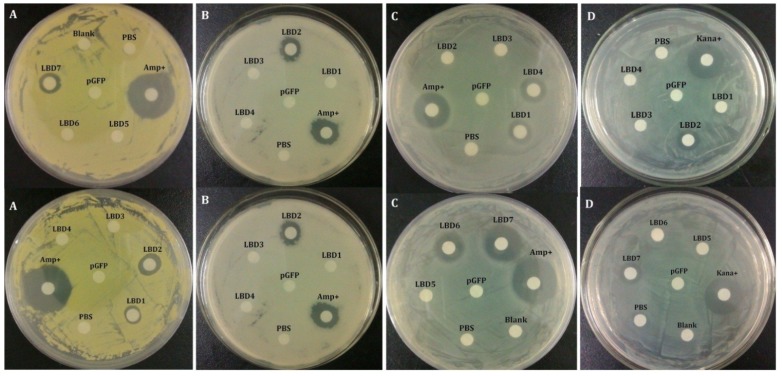
Inhibition zones of synthetic peptides corresponding to LBDs of different FcALFs. “Blank” represented blank group with nothing added. “PBS” represented control group with only PBS. “pGFP” represented negative control with synthetic pGFP peptide. “Amp+” and “Kana+” were positive controls with ampicillin and kanamycin sulfate separately. The bacterium strains *M. luteus* (**A**); *B. simplex* (**B**); *E. coli* (**C**); and *A. hydrophila* (**D**) were used for antibacterial analyses.

In order to know if FcALFs had antibacterial activities, inhibition zones and minimal inhibitory concentration (MIC) were examined using synthetic LBD peptides. Obvious inhibition zones with different sizes to *M. luteus* appeared around the filter paper that was loaded with LBD1, LBD2 and LBD3 peptide solutions, which correspond with that for positive controls of ampicillin (Amp+). For *E. coli*, LBD1, LBD4, LBD6 and LBD7-loaded paper also exhibited apparent inhibition zones. For *B. simplex*, similar inhibition zones were observed in LBD2, LBD5 and LBD7. Only LBD2 and LBD7-loaded paper exhibited inhibition zones in *A. hydrophila* plates. In addition, no inhibition zones appeared for negative control pGFP and blank control phosphate-buffered saline (PBS) either (Figure 2).

The antimicrobial activities of the synthetic LBD peptides were tested by measuring the MICs quantitatively. The inhibition of bacterial growth was considered when OD_600_ was significantly lower than that of PBS control. The data demonstrated that LBD1 effectively inhibited the growth of *M. luteus* and *E. coli* with the same MIC ranges of 16–32 μM. LBD2 could inhibit the growth of *M. luteus* and *B. simplex*, more effectively than *E. coli* and *A. hydrophila*. The MIC ranges of LBD2 peptide for *M. luteus* and *B. simplex* were 2–4 μM and 1–2 μM, respectively, while for *A. hydrophila*, the MIC was 32–64 μM. No antibacterial activity against four tested bacteria was observed for LBD3 peptide even at 64 μM. LBD4 and LBD6 were specifically against *E. coli* with the MIC ranges of 8–16 μM and 16–32 μM, respectively. LBD5 was specifically against *B. simplex* with the MIC range of 16–32 μM. Among the seven LBD peptides, LBD7 showed the most effective antibacterial activity with the broadest spectrum. It could inhibit the growth of both Gram-negative bacteria *E. coli* and *A. hydrophila* and Gram-positive bacteria *M. luteus* and *B. simplex*, especially against *M. luteus* and *A. hydrophila* with MIC ranges of 1–2 µM and 2–4 µM, respectively. The MICs of different peptides were shown in Table 1.

**Table 1:** Minimal inhibitory concentration of synthetic LPS-binding domain peptides on different bacterium strains.

Bacteria	MIC Range (μM)						
	LBD1	LBD2	LBD3	LBD4	LBD5	LBD6	LBD7
***M. luteus***	16–32	2–4	>64	>64	>64	>64	1–2
***B. simplex***	>64	1–2	>64	>64	16–32	>64	32–64
***E. coli***	16–32	>64	>64	8–16	>64	16–32	32–64
***A. hydrophila***	>64	32–64	>64	>64	>64	>64	2–4

### 2.3. Effects of Synthetic LBD Peptides of FcALFs on WSSV Infection

**Figure 3:**
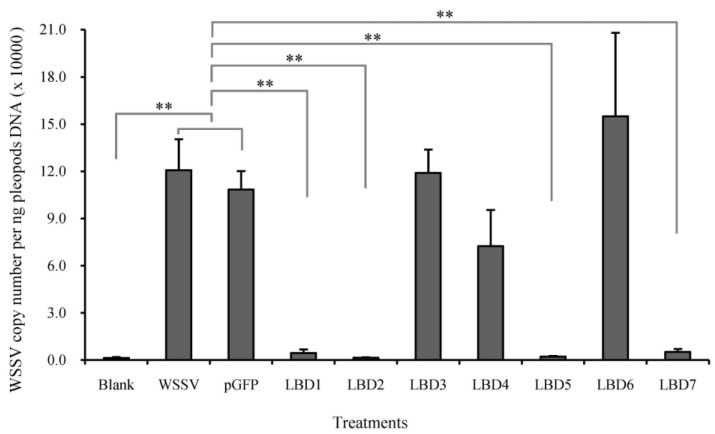
Comparison of viral propagation in *E. carinicauda* after infection with pre-incubated WSSV in synthetic peptides corresponding to LBDs of different FcALFs. “Blank” was group without WSSV or peptide injection and “WSSV” was group injected with WSSV. *n* = 15. Stars (**) indicated significant differences at *P* < 0.01 between different treatments.

In order to know if FcALFs could inhibit WSSV replication, WSSV pre-incubated with synthetic LBD peptides was injected into *E. carinicauda* and the *in vivo* WSSV copy number was tested. As shown in Figure 3, different LBD peptides showed distinct impacts on the WSSV infection. The background value of WSSV copy number in the blank group was 1.31 × 10^3^ copies/ng DNA. In WSSV group, the WSSV copy number reached 1.21 × 10^5^ copies/ng DNA, which had no significant difference with that in pGFP group (1.08 × 10^5^ copies/ng DNA). After incubation with LBD1, LBD2, LBD5 and LBD7, the copy numbers of WSSV in shrimp were obviously restricted. The WSSV copy number in LBD1, LBD2, LBD5 and LBD7 group were 4.36 × 10^3^ copies/ng DNA, 1.40 × 10^3^ copies/ng DNA, 2.07 × 10^3^ copies/ng DNA and 5.07 × 10^3^ copies/ng DNA, respectively, which were significantly lower than those in WSSV group and pGFP group. The WSSV copy number in LBD3, LBD4 and LBD6 group were 1.19 × 10^5^ copies/ng DNA, 7.24 × 10^4^ copies/ng DNA and 1.55 × 10^5^ copies/ng DNA, which had no significant differences with those in WSSV group and pGFP group.

### 2.4. Relationship between Antiviral Activity and the Sequence Feature of LBD Peptides

**Figure 4:**
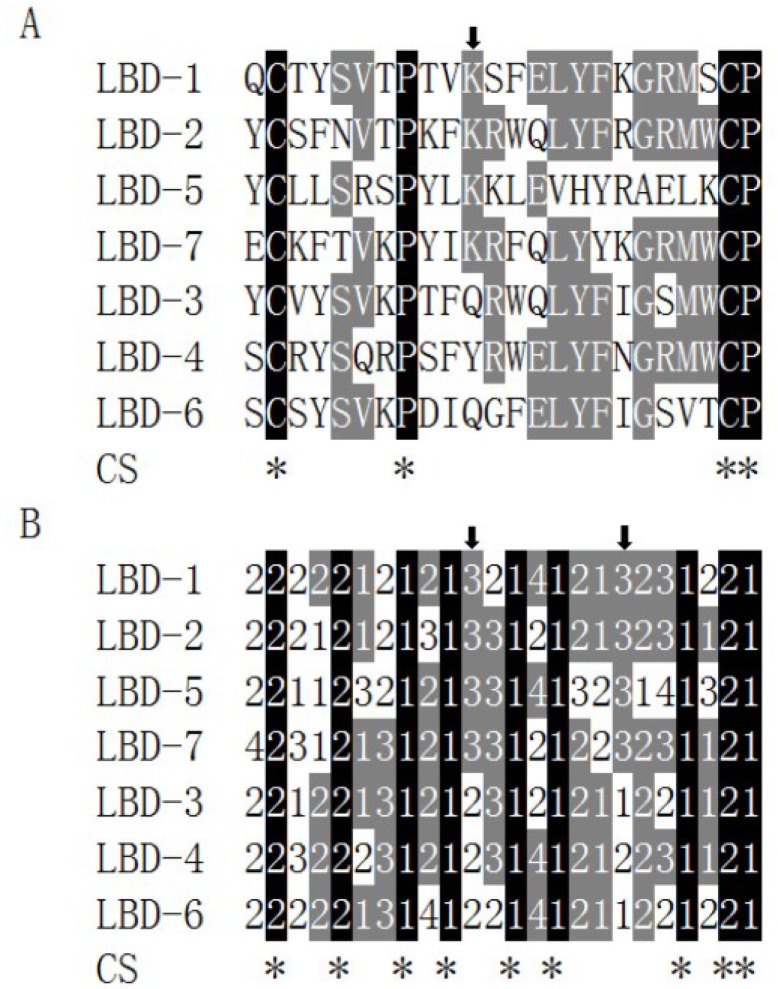
Sequence analyses of LBD peptides of different FcALFs. (**A**) Showed multiple alignments of LBD sequences from seven ALFs in *F. chinensis*; (**B**) Showed multiple alignments of these LBD sequences base on the acid-base properties of amino acid residues. “1”, “2”, “3” and “4” represented hydrophobic, neutral, basic and acidic amino acid residues, respectively. CS represented consensus sequence. Identical sites were marked by black background and shown with stars below. Sites with a similarity more than 50% were highlighted with gray background. Arrows showed identical sites that only existed in anti-WSSV peptides.

In order to find out if LBD peptides with anti-WSSV activities had similar characteristics, multiple alignments among them were performed. Data showed that sequence similarity among seven LBD peptides was very low and only three identical amino acid residues, two cysteine residues and one proline residue, were shared by them (Figure 4A). Taken the acid-base properties of the amino acid residues into consideration, higher similarity existed among seven LBD peptides, where a consensus sequence (2..2..1.1..1.1…..1.2, where 2 presented hydrophobic amino acid residues and 1 presented neutral amino acid residues) with eight identical sites were found (Figure 4B). One lysine site was identical among four LBD peptides with anti-WSSV activity, including LBD-1, LBD-2, LBD-5 and LBD-7, while not found at this site in other peptides (showed by an arrow in Figure 2A and the left arrow in Figure 4B). Besides, another identical basic amino acid site (showed by the right arrow in Figure 4B) only existed among four LBD peptides with anti-WSSV activity.

### 2.5. Effects of Modified LBD Peptides on WSSV Infection

**Figure 5:**
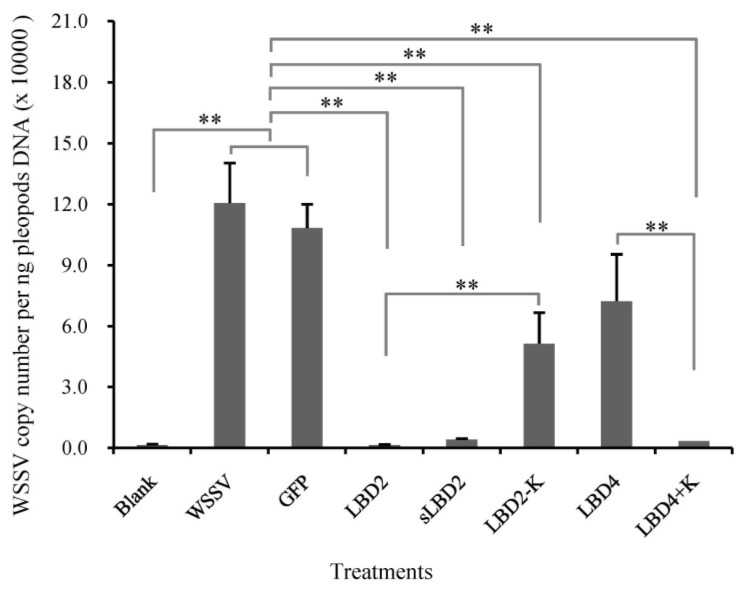
Comparison of viral propagation in *E. carinicauda* after infection with pre-incubated WSSV in modified peptides. Viral propagation was shown by measuring the expression level of VP28 in *E. carinicauda* at 24 h after infection of pre-incubated WSSV with 50 μM different peptides. “Blank” was group without WSSV or peptide injection and “WSSV” was group injected with WSSV. *n* = 15. Stars (**) indicated significant differences at *P* < 0.01 between different treatments.

From the sequence analysis results, we found that an identical lysine residue was important for the antiviral activity of LBD peptides. In order to verify the hypothesis, three new sequence-modified peptides were synthesized and named as sLBD2, LBD2-K and LBD4+K, respectively. The anti-WSSV effect of these peptides was detected and the result was shown in Figure 5. The WSSV copy number in sLBD2 treated group was 4.04 × 10^3^ copies/ng DNA, which was significantly lower than those in WSSV group and pGFP group, while no difference was found in sLBD2 group and LBD2 group. The WSSV copy number in LBD2-K treated group was 5.14 × 10^4^ copies/ng DNA, which was significantly higher than that in LBD2 group. The WSSV copy number in LBD4+K treated group was 3.40 × 10^3^ copies/ng DNA, which was significantly lower than those in WSSV group, pGFP group and LBD4 group.

## 3. Discussions

In the present study, we characterized seven types of ALF from the Chinese shrimp *F. chinensis*. Two of them, *FcALF2* and *ALFFc*, have been reported in our previous studies [22,23]. Here we characterized and compared the distribution and activities of all ALF isoforms in *F. chinensis* in detail.

Tissue distribution of the gene is always related with its biological function [28]. Seven ALFs exhibited diverse tissue distribution patterns and various antimicrobial properties. It was noticeable that all ALFs displayed high expression level in the lymphoid organ (Oka). *FcALF5*, *FcALF6* and *ALFFc* also showed high expression level in hemocytes, while *FcALF2*, *FcALF4* and *FcALF5* were also detected at a high expression level in eyestalk and *FcALF1* was highly expressed in stomach. Oka is deemed to play an important role in the innate immune system [29], and is the most specific and effective organ for clearance of bacteria [30] and virus [31] in penaeid shrimp. Hemocytes play important roles in the host immune response including recognition, phagocytosis, melanization, cytotoxicity and cell-cell communication [32]. Stomach was found as a target tissue for WSSV infection [33]. It is very curious that some ALF isoforms were highly expressed in eyestalk, which was always regarded as a neuroendocrine organ [34]. However, recent studies revealed that the eyestalk might contribute to crustacean immunity [24] and neuropeptides release from eyestalk to hemolymph was important for WSSV replication by elevating glycolytic activity and glucose mobilization [35]. A multiple and high expression patterns of *FcALFs* in these organs indicated that they might be participated in shrimp immune defense against distinct pathogens.

Previously, we found that FcALF2 exhibited strong inhibition activities against WSSV and Gram-positive bacteria, *M. luteus* and *M. lysodeikticus*, but poorly active against Gram-negative bacteria, *E. coli* and *V. anguillarum* [23]. However, ALFFc showed a quite different antimicrobial spectrum, which strongly inhibited WSSV, Gram-positive and Gram-negative bacteria [22]. In order to know the antimicrobial activities of other FcALFs, we synthesized the peptides corresponding to LPS-binding domains of these FcALFs. All synthetic peptides displayed antibacterial activities against one or more kinds of bacteria except LBD3, which showed no effect to the four tested bacteria species. Due to the limited number of tested bacteria species, it was not sure whether LBD3 inhibited the growth of other bacteria. It was also reported that different ALFs in other species showed distinct antimicrobial properties. In the swimming crab *P. trituberculatus*, six recombinant PtALF proteins could inhibit the growth of distinct Gram-positive, Gram-negative bacteria or Fungi [5,6,24,25]. The multiple antimicrobial properties of ALFs suggested that they might defense various pathogens synergistically in an organism.

ALF proteins were also reported to have antiviral activity. The recombinant ALF protein of *P. monodon* was found inhibiting herpes simplex virus type 1, human adenovirus respiratory strain and WSSV replication [10,11]. In our study, the LBD peptides from four of the FcALFs, including FcALF1, FcALF2, FcALF5 and ALFFc, could inhibit WSSV replication in experimental animals. Loss of antiviral function could be directly caused by replacing lysine residues with other amino acid residues in ALFFc-LBD peptide [22], which suggested that lysine residues in LBD peptides were important for their antiviral activities. However, increasing the number of lysine residues in LBD peptide did not show a better anti-WSSV performance [23], which might be due to the high peptides concentration (50 µM) concealed the difference among LBD peptides. Further sequence analysis discovered a conserved lysine residue site that only existed in LBD peptides with anti-WSSV activities. Interestingly, replacing this lysine residue with other amino acid residue in LBD peptide with anti-WSSV activity could lead to a considerable reduction of their anti-WSSV function, while replacing other residue with a lysine residue at this site in LBD peptide without anti-WSSV activity could endow them with anti-WSSV ability. The data proved that the certain lysine residue played a key role in inhibiting WSSV replication, which provided insight into understanding how ALFs exerted their anti-WSSV functions.

In conclusion, the present study compared the tissue distribution and antimicrobial activities of seven ALFs from the Chinese shrimp *F. chinensis*. Different ALFs possessed distinct antimicrobial activities. Sequence analysis and further function confirmation found that a certain lysine residue in LBD peptides is vital for their anti-WSSV activities. These results not only showed us a comprehensive understanding on the function of ALFs from *F. chinensis*, but also provided clues to development of ALFs as potential therapeutic drugs.

## 4. Materials and Methods

### 4.1. Animal and Tissue Collection

Healthy shrimp, *F. chinensis*, with a body weight of 38.97 ± 4.34 g, were acclimatized in the aerated seawater at 25 °C for three days before tissues collection. Hemolymph from ten individuals was collected from the ventral sinus located at the first abdominal segment with an equal volume of anticoagulant-modified Alsever solution (27 mM sodium citrate, 336 mM NaCl, 115 mM glucose, 9 mM EDTA, pH 7) [36]. Hemocytes from ten individuals were isolated by centrifugation at 800 g, 4 °C for 10 min and then preserved in liquid nitrogen. Other tissues including stomach, lymphoid organ (Oka), nerve cord, muscle, intestines, hepatopancreas, gill, heart, and eyestalk were dissected and preserved in liquid nitrogen respectively for RNA extraction and gene expression analysis.

*Exopalaemon* *carinicauda* is being regarded as a good crustacean model animal for WSSV infection experiments [37]. In this study, healthy *E. carinicauda* with a body length of 5.43 ± 0.30 cm and a body weight of 2.62 ± 0.42 g were used as experimental animals for WSSV infection assay. Animals were acclimatized in the aerated seawater at 25 °C for 1 day before experiments.

### 4.2. Total RNA Extraction, cDNA Synthesis and qPCR Analysis

The total RNA from each sample was extracted using Unizol reagent (BiostarGenechip, Shanghai, China). The first-strand cDNA was synthesized with M-MLV reverse transcriptase (Promega, Fitchburg, WI, USA) and oligodT primer.

The expression level of *FcALFs* in different tissues were detected by quantitative real-time RT-PCR with corresponding primers (Table 2) using a Master Cyclereprealplex (Eppendorf, Hamburg, Germany). The RT-PCR program was set as follows: 95 °C for 2 min, followed by 40 cycles of 94 °C for 20 s, annealing temperature for 20 s and 72 °C for 20 s. The expression level of 18S rRNA was used as an internal reference standard. The dissociation curve analysis of amplification products was performed at the end of each PCR reaction to confirm the specificity of the amplification. Then the data was analyzed by 2^−ΔΔ*C*^^T^ method [38] to calculate the relative gene expression levels.

### 4.3. Multiple Alignments of Synthetic LBD Peptides

Multiple alignments of synthetic LBD1, LBD2, LBD3, LBD4, LBD5, LBD6 and LBD7 peptides were carried out based on the sequences and the acid-base properties of the amino acid residues, respectively.

**Table 2:** Primers used for gene cloning and qPCR analysis.

Gene	Primer Name	Sequence (5′–3′)	Annealing Temperature (°C)
FcALF1	FcALF1-qF	ATGTCCTGCCCGTCCCTTAG	59
	FcALF1-qR	CCTCCGTTATCACGCCCTGT	
FcALF2	FcALF2-qF	TGCGAGTGTCAGTCTTTAGC	61
	FcALF2-qR	CAATCCTGTGAGTTTGTCCG	
FcALF3	FcALF3-qF	CAGGATTGTGGGAGACGGGA	61
	FcALF3-qR	CTGCTGCGTGTTTCGGCTAC	
FcALF4	FcALF4-qF	ACGATGCGAGTCTTGGTCAG	59
	FcALF4-qR	CTCATCCGAGTGCCACAACC	
FcALF5	FcALF5-qF	GCTTGTTGAGTCGCAGTCCT	59
	FcALF5-qR	GAGCCTGTCTTATGAAATCCTT	
FcALF6	FcALF6-qF	AGACTTATGGAGGAACGGAGAC	60
	FcALF6-qR	ATTTGCTGCGGGTGTTGGAC	
ALFFc	ALFFc-qF	AAGCCTGGTGCTGGTGGTGT	59
	ALFFc-qR	GAGTTCGGTTTTCTCGTTCCT	
18S rRNA	18S-qF	TATACGCTAGTGGAGCTGGAA	56
	18S-qR	GGGGAGGTAGTGACGAAAAAT	

### 4.4. Synthesis of Different LBD Peptides of FcALF Isoforms

Seven peptides corresponding to the LPS-binding domain (LBD) of different FcALF isoforms (Accession numbers of these genes were listed in Table 3) with one flanking amino acid residue in each N and C terminal were synthesized. They were named as LBD1-7, respectively. A disulfide bond was formed between two cysteine residues in each peptide. According to our previous studies [22,23], the peptides corresponding to LBDs of FcALF2 and ALFFc were used as positive control. Another peptide with 24 amino acid residues based on the amino acid sequence of Green Fluorescent Protein (pGFP) was also synthesized as a negative control. Based on the results of our functional experiments, another three peptides, LBD2-K, sLBD2 and LBD4+K, were also synthesized. The sequence of LBD2-K was the same as LBD2 except for the replacement of a lysine residue by a glutamine residue. The peptide sLBD2 was a part (ten amino acid residues) of LBD. The sequence of LBD4+K was the same as LBD4 except for the replacement of a tyrosine residue by a lysine residue. All peptides were acetylated in the N terminal and amidated in the C terminal. These peptides were all synthesized in Ziyu Biotechnology Co. Ltd. (Shanghai, China). The detailed sequence information of above synthesized peptides was shown in Table 3.

### 4.5. Inhibition Zone Test

Gram-negative bacteria, including *Escherichia coli* and *Aeromonas hydrophila*, and Gram-positive bacteria, including *Micrococcus luteus* and *Bacillus simplex* were used for antibacterial analyses. The strains were incubated in fresh LB medium overnight at 37 °C for *E. coli*, *M. luteus* and 28 °C for *A. hydrophila*, *B. simplex*.

**Table 3:** Sequence information of synthetic peptides.

Gene Name	Accession Number	Peptide Name	Sequence
FcALF1	JX853774	LBD1	Ac-Q(CTYSVTPTVKSFELYFKGRMSC)P-NH_2_
FcALF2	JX853775	LBD2	Ac-Y(CSFNVTPKFKRWQLYFRGRMWC)P-NH_2_
FcALF3	JX853776	LBD3	Ac-Y(CVYSVKPTFQRWQLYFIGSMWC)P-NH_2_
FcALF4	JX853777	LBD4	Ac-S(CRYSQRPSFYRWELYFNGRMWC)P-NH_2_
FcALF5	JX853778	LBD5	Ac-Y(CLLSRSPYLKKLEVHYRAELKC)P-NH_2_
FcALF6	JX853779	LBD6	Ac-S(CSYSVKPDIQGFELYFIGSVTC)P-NH_2_
ALFFc	AY859500	LBD7	Ac-E(CKFTVKPYIKRFQLYYKGRMWC)P-NH_2_
/	/	LBD2-K	Ac-Y(CSFNVTPKFQRWQLYFRGRMWC)P-NH_2_
/	/	sLBD2	Ac-FKRWQLYFRG-NH_2_
/	/	LBD4+K	Ac-S(CRYSQRPSFKRWELYFNGRMWC)P-NH_2_
GFP	AAN41637	pGFP	Ac-TTGKLPVPWPTLVTTFSYGVQCFS-NH_2_

Note: “Ac-”represents acetylation of the *N*-terminal amino acid residue; “–NH2” represents amidation of the *C*-terminal amino acid residue; parentheses at two sides of Cysteine amino acids shows a disulfide bond.

The antibacterial activities of LBD peptides were examined by inhibition zone test. The overnight cultures of bacteria were diluted 100 times and cultured in LB medium at 37 °C (*E. coli* and *M. luteus*) or 28 °C (*A. hydrophila* and *B. simplex*) for 6 h. These cultures were then diluted 100 times with LB medium separately, and 200 μL solutions from each diluted cultures was taken out and spread on the solid LB medium plate uniformly. Sterile filter paper with a diameter of 6 mm was put on the surface of solid LB medium gently. Twenty microliters of 64 μM peptide solution dissolved in PBS (pH 7.4) was added to the center of filter paper with a diameter of 6 mm. Twenty microliters PBS and 20 μL of 64 μM pGFP peptide were used as negative controls. Fifty microliters ampicillin solutions was used as the positive control for *E. coli*, *M. luteus* and *B. simplex* culture, and 50 μM kanamycin sulfates was used as the positive control for *A. hydrophila* culture. The plates were cultured at 37 °C (*E. coli* and *M. luteus*) or 28 °C (*A. hydrophila* and *B. simplex*) for 24 h.

### 4.6. Minimal Inhibitory Concentration (MIC) Assay

MIC assay was performed according to the method described by Wiegand *et al*. [39] with slight modification. The bacterial strains were culture in LB medium at 37 °C (*E. coli* and *M. luteus*) or 28 °C (*A. hydrophila* and *B. simplex*) till the OD_600_ reached certain values corresponding to ~1 × 10^8^ colony forming units (CFU)/mL. Then, 2 μL of the bacterial cultures, 20 μL of 1/2-fold serially diluted peptides (640 μM–10 μM) in PBS (pH 7.4), and 178 μL of fresh LB medium were added into each well of sterile 48-well plates. The final concentration of the bacterial cultures was diluted to ~1 × 10^6^ CFU/mL. The peptides solutions were diluted at 1/2-fold serial concentrations from 64 μM to 1 μM in a final volume of 200 μL. The 48-well plates were incubated at 37 °C (*E. coli* and *M. luteus*) or 28 °C (*A. hydrophila* and *B. simplex*) for 3 h. Then, 300 μL of fresh LB medium was added and the plates were incubated at the corresponding temperatures for another 18 h. The optical density (OD) was measured at 600 nm (OD_600_) using a micro-plate reader (TECAN infinite M200 PRO, Austria). PBS and pGFP at the same concentration with LBD peptides were used as negative controls. Antibiotics (50 μM ampicillin for *E. coli*, *M. luteus*, and *B. simplex*, 50 μM kanamycin for *A. hydrophila*) were used as positive controls. The assay was performed in triplicates.

### 4.7. Antiviral Activity Detection

In order to learn the effect of synthetic LBD peptides on WSSV infection, WSSV pre-incubated with different peptides was injected into *E. carinicauda* and the WSSV copy number in the pleopods of *E. carinicauda* was detected. *E. carinicauda* was classified into 13 groups and each group contained 18 to 20 individuals. The 13 groups were named as Blank, WSSV, pGFP, LBD1, LBD2, LBD3, LBD4, LBD5, LBD6, LBD7, sLBD2, LBD2-K and LBD4+K, respectively. Synthetic peptides were diluted into 50 μM with PBS (pH 7.4). WSSV was incubated with each peptide solution for 2 h at room temperature with a final concentration 10^3^ copies/μL. For Blank group, shrimp did not suffer any injection. For WSSV group, each shrimp was injected with 10 μL WSSV incubated with PBS (pH 7.4) for 2 h at room temperature. For the other groups (LBD1–LBD7, sLBD2, LBD2-K and LBD4+K), each shrimp was injected with 10 μL of WSSV solution after incubation with corresponding synthetic peptides, respectively. At 24 h post injection (hpi), the pleopods of 15 shrimps in each group were collected and the pleopods from three individuals were put together as one sample. Total DNA of each sample was extracted using the Genomic DNA Kit (Tiangen, China). The viral copy number was measured by detecting the level of VP28 using real-time PCR as described previously [37].

### 4.8. Statistical Analysis

All data were given in the form of mean ± SE and analyzed with one-way analysis of variance (ANOVA) and Ducan’s Multiple Comparisons. Differences between treatments and controls were considered significant at *p* < 0.05.
